# Improving Transplant Medication Safety Through a Pharmacist-Empowered, Patient-Centered, mHealth-Based Intervention: TRANSAFE Rx Study Protocol

**DOI:** 10.2196/resprot.9078

**Published:** 2018-03-02

**Authors:** James N Fleming, Frank Treiber, John McGillicuddy, Mulugeta Gebregziabher, David J Taber

**Affiliations:** ^1^ Department of Pharmacy Medical University of South Carolina Charleston, SC United States; ^2^ Technology Center to Advance Healthful Lifestyles College of Nursing Medical University of South Carolina Charleston, SC United States; ^3^ Department of Surgery Medical University of South Carolina Charleston, SC United States; ^4^ Department of Public Health Sciences Medical University of South Carolina Charleston, SC United States; ^5^ Department of Pharmacy Ralph H Johnson Veterans Affairs Medical Center Charleston, SC United States

**Keywords:** telemedicine, mhealth, transplant, clinical trial, errors, adherence

## Abstract

**Background:**

Medication errors, adverse drug events, and nonadherence are the predominant causes of graft loss in kidney transplant recipients and lead to increased healthcare utilization. Research has demonstrated that clinical pharmacists have the unique education and training to identify these events early and develop strategies to mitigate or prevent downstream sequelae. In addition, studies utilizing mHealth interventions have demonstrated success in improving the control of chronic conditions that lead to kidney transplant deterioration.

**Objective:**

The goal of the prospective, randomized TRANSAFE Rx study is to measure the clinical and economic effectiveness of a pharmacist-led, mHealth-based intervention, as compared to usual care, in kidney transplant recipients.

**Methods:**

TRANSAFE Rx is a 12-month, parallel, two-arm, 1:1 randomized controlled clinical trial involving 136 participants (68 in each arm) and measuring the clinical and economic effectiveness of a pharmacist-led intervention which utilizes an innovative mobile health application to improve medication safety and health outcomes, as compared to usual posttransplant care.

**Results:**

The primary outcome measure of this study will be the incidence and severity of MEs and ADRs, which will be identified, categorized, and compared between the intervention and control cohorts. The exploratory outcome measures of this study are to compare the incidence and severity of acute rejections, infections, graft function, graft loss, and death between research cohorts and measure the association between medication safety issues and these events. Additional data that will be gathered includes sociodemographics, health literacy, depression, and support.

**Conclusions:**

With this report we describe the study design, methods, and outcome measures that will be utilized in the ongoing TRANSAFE Rx clinical trial.

**Trial Registration:**

ClinicalTrials.gov NCT03247322: https://clinicaltrials.gov/ct2/show/NCT03247322 (Archived by WebCite at http://www.webcitation.org/6xcSUnuzW)

## Introduction

Kidney transplantation is considered the preferred treatment option for patients with end-stage renal disease, with more than 140,000 patients living in the U.S. with a functioning transplant [1]. The use of potent contemporary immunosuppression has significantly decreased acute rejection rates, with current one-year rates of <10%, compared to 30%-40% three decades prior [2-4]. Despite this, long-term renal allograft survival remains largely unchanged during this time period. Studies have demonstrated that predominant causes of graft loss are driven by immunosuppression adverse drug events (ADE) (patient harm related to a medication) and rejection from medication nonadherence (MNA) [5-7]. These origins of graft loss encompass issues directly related to medication safety. Current immunosuppression regimens are highly effective but carry the burdens of considerable toxicities and exceeding complexity [8]. These attributes place a transplant patient at high risk of developing ADEs and medication errors (ME). Several studies suggest that ADE, particularly surrounding infection from over-immunosuppression and calcineurin inhibitor nephrotoxicity, may be a predominant cause for the discordance noted between reductions in acute rejection and lack of improvements in graft survival. In 2006, Parasuraman, et al. demonstrated that infectious etiologies surpassed rejections as the leading cause of death-censored graft lost [7]. Despite this, there are limited studies analyzing the incidence, etiologies, and outcomes associated with medication safety issues [9,10].

Our formative research has demonstrated that MEs (taking a med in a manner not intended), predominantly due to patient-related factors, occur in nearly two-thirds of kidney transplant recipients, leading to hospitalization in 1 out of every 8 recipients [11,12]. Further, we found that recipients that develop clinically significant MEs are at considerably higher risk of deleterious clinical outcomes, most significantly graft loss; these patients also develop substantially more ADEs, readmissions, and acute rejections [11,12]. We have also demonstrated that immunosuppressant ADEs are correlated with MEs; patients that experience MEs leading to hospitalization have 2.3 times the risk of developing at least three ADEs (*P*=.02, Table 1) [12]. In other chronic disease states, ADEs have clearly been established as a major risk factor for MNA [13-15]. Therefore, early recognition of ADEs in kidney transplant recipients will likely help prevent downstream clinical sequelae, including MNA and irreversible immunosuppressant toxicities. Research demonstrates that clinical pharmacists have the unique education and training to identify these events early, as well as developing strategies to mitigate or resolve the associated sequelae [16-21].

### Objectives

Due to the complexities and toxicities associated with their immunosuppressive medication regimens, kidney transplant recipients are at high risk of developing medication safety issues which can lead to hospitalization, increased healthcare expenditures, and ultimately graft loss. Founded on preliminary information, the use of pharmacists and mobile health (mHealth) technology provide a promising and innovative approach to improve medication safety in high-risk patients. The ultimate goal of this research is to demonstrate how patients, pharmacists, and technology can work hand-in-hand to optimize medication-related outcomes and reduce healthcare expenditures.

## Methods

### Study Design

TRANSAFE Rx is a 12-month, parallel, two-arm, 1:1 randomized controlled clinical trial involving 136 participants (68 in each arm) and measuring the clinical and economic effectiveness of a pharmacist-led intervention which utilizes an innovative mHealth application to improve medication safety and health outcomes, as compared to usual post-transplant care. This study has been approved by the local Institutional Review Board (IRB) and conforms to the clinicaltrial.gov guidelines.

### Aims

The primary aim of the TRANSAFE Rx study is to compare the incidence, severity, and etiologies of MEs and ADEs in kidney transplant recipients under normal care with recipients randomized to a pharmacist-led innovative mHealth intervention. Secondarily, we will compare the total resources utilized to provide care between the cohorts and measure the impact of MEs and ADEs on clinical outcomes.

### Recruitment, Screening, and Enrollment Procedures

Adult (≥18 years old at the time of transplant) solitary kidney transplant recipients 6 to 36 months posttransplant that meet study eligibility will be identified through review of patients visiting the kidney transplant clinic as part of usual care and approached by research personnel for consideration for participation. Patients will be required to complete an informed consent document to ensure they understand the goals, risks, and potential benefits of the study before any research related activities occur. Patients will be randomized into one of the two groups by random selection using a random number generator in a simple blocked manner (blocks of 8). Due to the nature of the intervention, complete blinding of the subject and research staff is unable to be performed. In order to minimize bias, data for MEs, ADEs and clinical outcomes will be collected by blinded study coordinators and clinical pharmacists.

### Eligibility

#### Inclusion criteria

Participants must be adult (≥ 18 years of age) kidney transplant recipients between 6 and 36 months posttransplant and their primary transplant physician must agree that they may participate.

#### Exclusion criteria

We will exclude multi-organ transplant recipients and any patient that is incapable of measuring their own blood pressure and blood glucose (if applicable); self-administering medications; speaking, hearing and reading English; or utilizing the mHealth application after sufficient training.

**Table 1:** Televisit schedule based on patient risk.

Risk Level	Definition	Scheduled Televisits	Triggered Televisits
High	Meets 2 or more of the following High-Risk Criteria: <80% adherence to medications Missed clinic visits Blood pressure outside of 20% of goal <80% of blood sugars within goal range Moderate to severe side effects	Twice Monthly	Patient-reported medication change or initiation New severe medication side effect Critical home values of blood pressures or glucoses Any transition in care
Moderate	Meets 1 of the High-Risk Criteria	Monthly	
Low	Does not meet any of the High-Risk Criteria	None Necessary	

### Sample Size Requirements

Based on previous studies conducted by our research collaborative, we estimate that approximately 64% of kidney transplant recipients in the control group will experience at least one ME during the one year study (defined using the Overhage criteria) [12,17]. Our previous research demonstrates that pharmacist-led initiatives can reduce these MEs by approximately 50% [14,15]. Using these estimates, enrolling 104 participants (52 in each cohort), will provide 92% power in detecting a statistically significant difference in ME event rates, with a two-tailed α=0.05. We will also have 94% power (two-tailed, α=0.05) to detect a 33% reduction in significant ADEs (CTCAE grade 3 or higher), given an estimated incidence rate of 87% in the control cohort and the strong association between MEs and ADEs [12-15]. From previous analyses, we expect that the control cohort will have a mean of 18.4 (SD 2.6) healthcare encounters (clinic visits, acute care/ER visits and hospitalizations) during the one year study. We estimate the intervention group will see an 8% absolute reduction in total encounters, to a mean 17.0 encounters, with an estimated 33% relative reduction in the mean number of hospital readmissions (1.2 vs 0.8, respectively). A previous study demonstrated a 47% reduction in 30-day readmission rates with a pharmacist-led intervention that improved admit and discharge medication reconciliation accuracy [16]. Although the current study employs a different intervention in a population at lower risk of readmission, we estimated that we could expect a 33% reduction in hospital readmissions within a year based on the previous data. Given these estimates (two-tailed, α=0.05), enrolling 52 patients in each arm will provide 80% power to detect a statistically significant difference. It is estimated that the intervention will also produce a mean cost savings of at least US $2489 per patient (US $7658 in the control cohort and US $5169 in the intervention cohort, with a SD estimated at US $4,530) [12]. This study is expected to have >80% power to detect a statistically significant difference in total posttransplant costs between cohorts, given these estimates.

For the exploratory outcomes of acute rejection, infections, graft function, graft loss, and death, this study is not powered to detect statistically significant differences in these clinical events between groups. However, we expect to demonstrate meaningful clinical signals, particularly with a reduction in acute rejection. Our previous study demonstrated an acute rejection rate that was 1.8 times higher in patients experiencing a significant medication error (13.7% vs 7.7%, respectively) [11]. Thus, we expect an overall acute rejection rate of 12% in the control cohort and 9% in the intervention cohort, corresponding with a 25% relative reduction in acute rejection rates. Based on previous randomized controlled trials conducted within the study institution, we expect to maintain an 85% retention rate. We adjusted our total sample size to 136 patients (68 in each cohort) to account for dropouts, thus maintaining adequate sample size to produce at least 80% power to detect statistically significant differences in the primary outcome measures.

### Intervention

Patients randomized to the intervention cohort will be provided the same usual care as the control cohort. In addition, this cohort of participants will receive clinical pharmacist-led supplemental medication therapy monitoring and management, utilizing a smartphone-enabled mHealth app, integrated with televisits and home-based monitoring of blood pressures and glucoses (when applicable). Patients in this cohort will be provided with a mobile device/data plan if they are not current owners of an iPhone version 4 or later (Apple, Cupertino, CA). All will also be provided with a Bluetooth-enabled, automated, cuff-style bicep home blood pressure monitor and a Bluetooth-enabled digital home blood glucose monitor (if the patient has diabetes; ForaCare, Moorpark, CA). On the mobile device, a HIPPA compliant app developed by our collaborative group will be installed that displays the patient’s medication list and alerts them when it is time to take each medication, requiring them to indicate if the medication was taken for adherence tracking. Through the app, medication regimen-specific symptom surveys will be pushed to patients that ask the frequency and severity of common side effects of their medications on a monthly basis and on-demand by the subject. The intervention will include a clinical transplant pharmacist telemonitoring subject medication; medical appointment adherence; weekly blood pressure/glucose readings (if applicable); and scheduling telehealth visits with patients, as outlined in Table 1.

**Figure 1:**
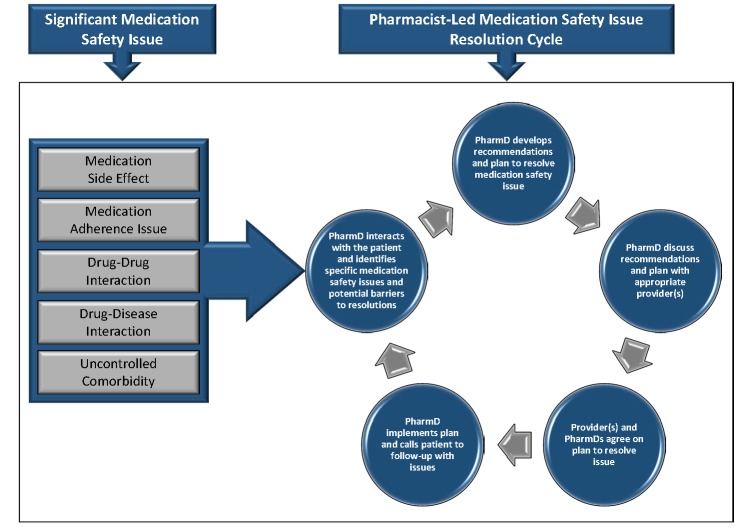
Pharmacist process to resolve medication safety issues.

**Figure 2:**
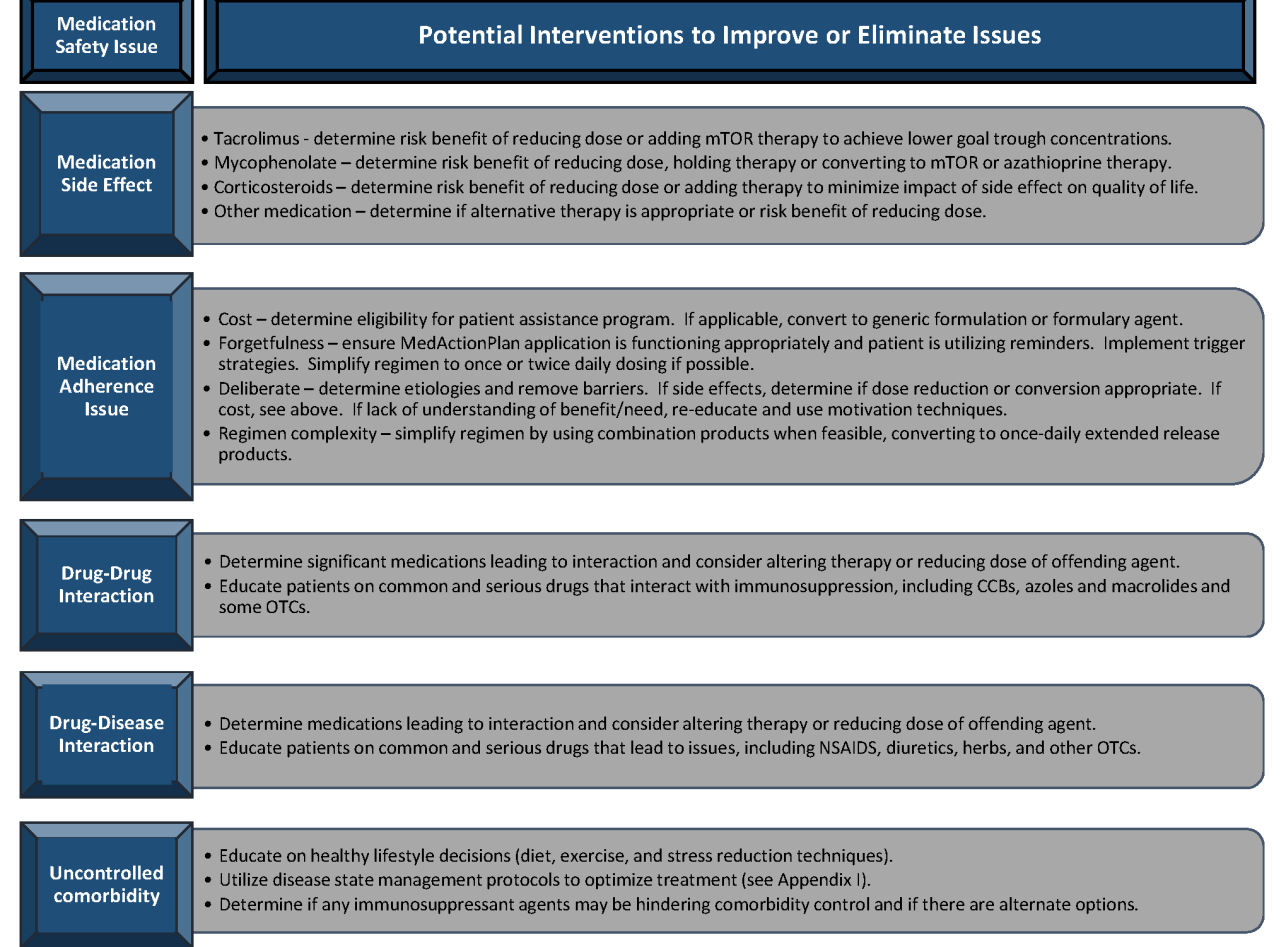
Predominant medication safety issues with mitigating interventions.

The clinical transplant pharmacist will be alerted by the patient if there are medication changes made by outside providers, through a patient-initiated notification on the app in addition to prescription refill monitoring (SureScripts, Arlington, VA). At this point, the patient will be contacted to evaluate the medication change and determine if the adjustment to the regimen is safe and effective. If the pharmacist deems this change to be of concern, they will work with the patient and prescribing physician to alter the regimen in an appropriate manner. In addition, the pharmacist will be alerted if the patient has evidence of significant nonadherence (≥20% missed self-reported medication doses in the course of a week), if they have blood pressure or glucose values that fall into critical ranges or if there are alarming trends in their readings or symptom assessments from surveys. Upon receiving these alerts, the pharmacist will communicate with the patient, determine the root cause, and coordinate care with other care providers as delineated at the bottom of Figure 1. During televisit encounters, the transplant pharmacist will conduct a thorough medication review, evaluate for signs and symptoms suggestive of medication safety issues, screen for drug-drug and drug-disease interactions and provide recommendations to resolve identified issues to the patient and/or provider, when applicable. The clinical pharmacist will be alerted and evaluate each patient when making a transition of care (emergency room visit, inpatient admission or discharge) to ensure accurate medication regimens are communicated to accepting teams and to the patient. The process used to resolve medication safety issues during distant monitoring is outlined in Figure 2. Once the clinical pharmacist identifies an issue, they will develop a management plan using the algorithm detailed in Figure 2, discuss the recommendations with the providers, agree on a plan, and implement the plan with direct patient follow-up. The algorithm in Figure 2 encompasses the major medication safety issues, including side effects, adherence, drug interactions and less than optimally controlled comorbid disease states. This algorithm is a guideline, and the transplant pharmacist will use this, as well as their clinical judgment and professional experience, to develop the medication safety issue resolution plan.

#### mHealth Medication Safety Monitoring and Management Tool

**Figure 3:**
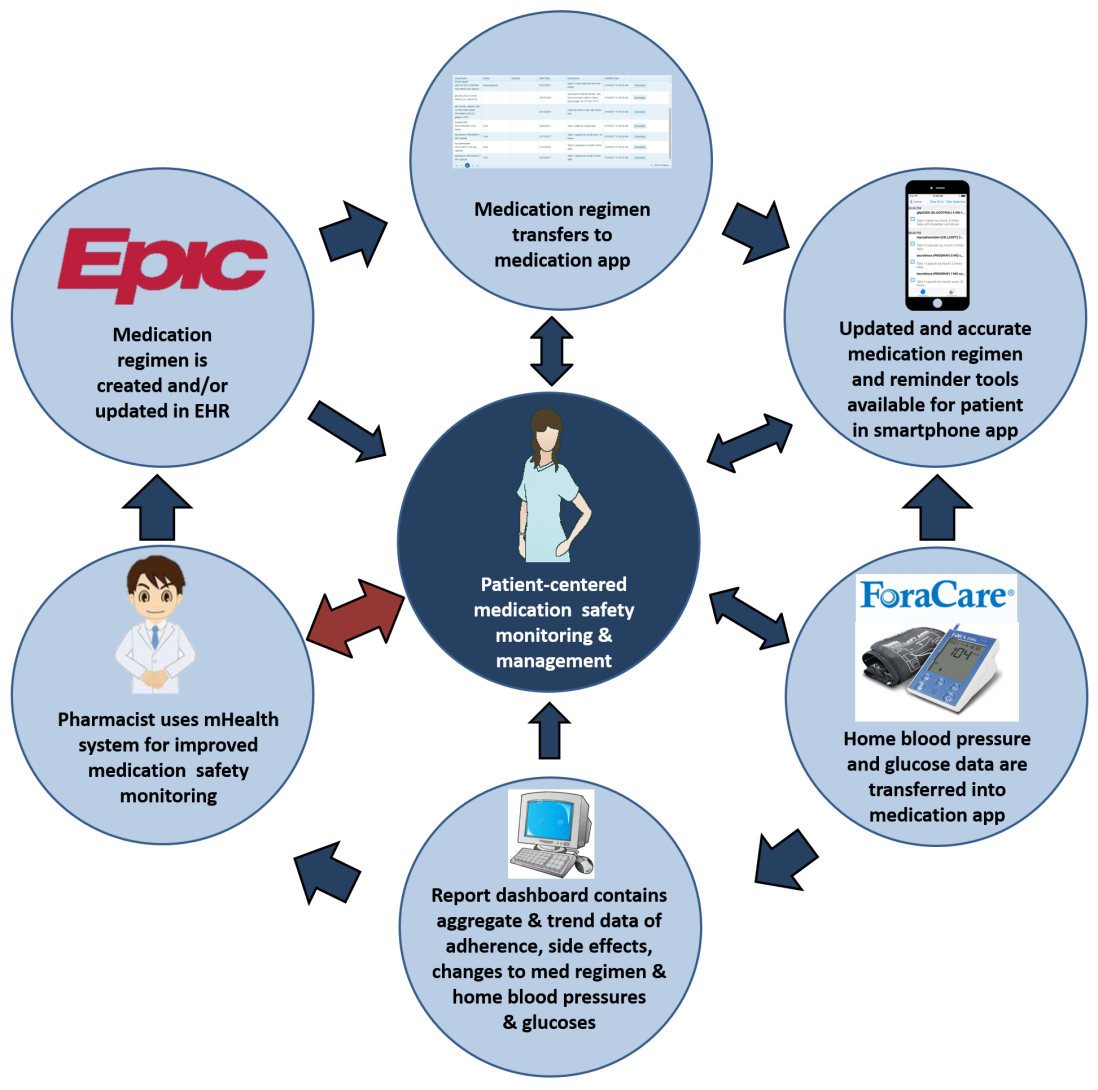
Conceptual diagram of mobile health (mHealth) system.

Patients in the intervention cohort will have enhanced medication safety monitoring utilizing an integrated mHealth system, coalescing the EHR (EPIC, Verona, WI) with an application developed by our research collaborative and FORACare telehealth systems to provide a seamless, bidirectional, patient-centered, home-based monitoring tool that will allow for early, effective, and efficient identification of medication safety issues by the clinical transplant pharmacist. The application will provide patients with useful tools to conduct self-care monitoring and management, including timely reminders to take medications; automated messages when patients miss multiple medication doses or scheduled health monitoring; tracking of medication side effects; and reporting of trends in blood pressures and glucoses (when applicable). Using our foundational research and through previous collaborations, we have partnered with Technology Applications Center for Healthful Lifestyles (TACHL) to incorporate monitoring tools and patient questionnaires that will minimize intrusions, while maximizing the potential of identifying medication safety issues, including MEs, nonadherence and ADEs, early in their course (Figure 3).

### Safety Monitoring

The data safety monitoring plan (DSMP) includes the use of a safety officer, with overarching IRB oversight, to monitor the study-related clinical outcomes, medication side effects, and adverse events. Additionally, the DSMP will utilize the study statistician to review the data generated by the TRANSAFE Rx study and ensure data integrity. Summaries of adverse event reports and patient safety concerns raised by the safety officer will be made to AHRQ in yearly progress reports unless the nature of a particular event is such that it bears reporting to the NIH immediately.

Both the safety officer and the biostatistician will coordinate data review and analysis and work closely with the study Principle Investigator (PI) and the co-investigators. The functions of the designated safety officer are to: 1) provide scientific oversight; 2) review all adverse effects or complications related to the study; 3) monitor accrual; 4) review summary reports relating to compliance with protocol requirements; and 5) provide advice on resource allocation.

The safety officer and statistician will meet at the following seven predesignated study milestones: each time 34 patients have received at least 6 months of study follow-up care (four meetings); once 68 patients have completed the study (one meeting); once 102 patients have completed the study (one meeting); and at study close-out. The team will also meet on an as-needed basis for any unexpected serious adverse events or significant study findings. Data will be provided at these meetings by the investigators on key variables that may indicate harm, including significant medication safety events leading to hospitalization or intervention. Study patient clinical events, including hospitalizations, emergency room visits, acute rejections, life-threatening infections, graft loss and patient death will also be reviewed during these sessions. The biostatistician will evaluate confidentiality and integrity of the database, and the procedures for recording and storing confidential files. The safety officer will also review the elements of the research plan to deal with emergencies. At the conclusion of these meetings, the recommendations of the safety officer will be reviewed and the PI and co-investigators will take appropriate corrective actions as needed.

The safety officer will have the authority to halt the trial if he/she perceives that harm is occurring due to the interventions.

### Intervention Protocol Adherence

Monitoring for adherence with medications, clinic visits, blood pressure and glucose monitoring (if applicable) is a part of the intervention in the intervention cohort. If patients are not adhering to data monitoring in the intervention cohort, they will be contacted by the study pharmacist to encourage use of the smartphone and any relevant Bluetooth-enabled devices. If this does not resolve the nonadherence, the subject will be contacted by the study PI to discuss continued involvement in the study. These data will not be monitored real-time in the usual care cohort.

In both cohorts, adherence with study data capture will be monitored by the study coordinators, who are completely independent of the pharmacists providing the interventions. Data that will be gathered by the coordinators includes blood pressures and laboratories measured at clinic visits, number of clinic visits, hospital admissions, infections, rejections, graft loss, and number and type of medication errors. They will gather data via direct subject interview and through review of the subject’s electronic medical records.

### Statistical Analysis Plan

This analysis will incorporate the intent-to-treat principle, namely, all randomized participants will be included in the analysis according to their intervention assigned at baseline. The two groups will be compared using standard statistical analyses. Data will be reported using percentages for nominal and ordinal variables and compared using Fisher’s exact test or Pearson’s chi-squared test as appropriate. This includes baseline demographic and transplant characteristic variables, as well as the outcome variables of the incidence and severity of medication errors and adverse drug events, acute rejection, and infections. For continuous variables with normal distribution, results will be reported using means and SDs with statistical comparison using Student’s *t* test for two independent samples. For nonnormally distributed variables, the results will be reported using medians and interquartile ranges, with statistical comparison conducted using the Mann-Whitney U test. Normal distribution of continuous variables will be assessed using normality plots and the Shapiro-Wilk test. Normal variance will be assessed using Levene’s test for equality of variances. Results for graft and patient survival will also be reported using Kaplan-Meier survival curves and compared using the Log Rank test.

If it is determined that there are significant imbalances in baseline demographics or characteristics known to influence any of the outcome measures, multivariable modelling will be used to adjust for these differences. For nominal outcomes, binary logistic regression will be used in a standard entry fashion, which will include both the grouping variable and all known risk factors. For continuous outcomes that demonstrate linearity in the relationship between dependent and independent variables, with a lack of serial correlation between covariates, homoscedasticity of the errors, and normality of the error distribution, linear regression will be utilized in a similar manner. We will adjust for baseline values if the interventions are discrepant at baseline. This model will include the intervention arm and baseline response as fixed effects and is known to lead to very precise inference [22]. If any of the four aforementioned assumptions are violated, then the data variables will either be transformed or appropriate substitute multivariable modelling will be used. Cox proportional hazard regression analysis will be used for time-dependent survival analyses involving the outcomes of graft and patient survival. For count outcomes, such as health care encounters, we will use Poisson regression; if the assumption of equal mean and variance is violated (over dispersion), we will use Negative Binomial regression. In all models, we will adjust for correlation of outcomes by including random effect terms. For all models that belong to the generalized linear model (linear, logistic, Poisson), we will use generalized estimating equations, and for survival outcomes, we will use frailty Cox regression [23]. We will use multiple imputation techniques to deal with missing data that is at random (MAR) [24]. MAR assumes that the probability that an outcome is missing depends on observed outcomes. While mechanisms for missingness are likely to be MAR, we will also do sensitivity analysis for data missing not at random (MNAR) using methods from Little and Rubin [24].

## Results

The primary outcome measure of this study will be the incidence and severity of MEs and ADRs, which will be identified, categorized, and compared between the intervention and control cohorts. The exploratory outcome measures of this study are to compare the incidence and severity of acute rejections, infections, graft function, graft loss, and death between research cohorts and measure the association between medication safety issues and these events. Additional data that will be gathered includes sociodemographics, health literacy, depression, and support. These are important variables that may modify or confound the impact of the intervention.

### Study Endpoint Definitions and Assessment Plan:

The following will be used to define and capture data and events within this study:

1. MEs are defined as documentation that a patient is taking a medication in a manner that was not intended; synonymous with the definition developed by Overhage and utilized within our previous research [12,17].
2. ADEs are defined according to the AHRQ Patient Safety Network, in which it describes an adverse drug event as “an adverse event (ie, injury resulting from medical care) involving medication use” [25]. The severity of the ADE will be defined according to a modified version of the CTCAE developed by the National Cancer Institute and utilized in our previous research. In both the usual care and intervention arms, a highly trained clinical research coordinator will independently interview all participants at bimonthly intervals and review their medical records to capture and record all MEs and ADEs, including timing, likely cause, and severity of each event. To assess for ME, the research coordinator will review and compare the patient’s documented medication regimen in the electronic health record (the regimen intended to be taken) to the medication regimen actually being taken by the patient. To assess for ADEs, the research coordinator will review patient symptomology, vital signs and laboratory values.
3. Acute rejection will be defined as a renal allograft biopsy demonstrating at least grade 1A rejection by Banff ’97 criteria or higher or treated borderline rejection [26]. All patients will be required to have biopsy confirmation of rejection episodes within 24 hours of onset of treatment for acute rejection, as per our protocol and usual care. It is standard care that all kidney allograft biopsies performed for transplant recipients occur at the transplant center (study institution). Biopsies will be read by the local pathologist, as per usual care. This pathologist will not be informed of participant participation in the study and will be blinded to cohort assignment. The study coordinator capturing clinical event data, different from the screening and randomizing coordinator (to ensure blinding is maintained), will review the medical record at regular intervals to determine the incidence, timing, severity, treatment regimen, and reversibility of each acute rejection episode for all study participants.
4. Infections will be defined as any diagnosed and treated infection, and will be subclassified as bacterial, viral, or fungal etiologies. Flu-like illnesses and viral syndromes NOT requiring antimicrobial therapy will not be defined as infections for this study. Opportunistic infections will also be subclassified for this study as viral, bacterial or fungal and defined as infections not seen in immunocompetent individuals; the most common opportunistic infections in kidney transplant recipients include cytomegalovirus (CMV), BK virus, Ebstein-Barr virus (EBV) and candidiasis [27,28]. The study coordinator capturing clinical event data will review the medical record at regular intervals to determine the incidence, timing, severity, treatment regimen, and cure timing of each infection episode for all study participants.
5. Graft function will be defined using the 4-variable Modification of Diet in Renal Disease equation to estimate glomerular filtration rate (GFR). This equation has been validated as an accurate reflection of true GFR within kidney transplant recipients [29]. Routine serum creatinine concentrations, which are measured as part of usual care, will be utilized to estimate GFR at these approximate time points: baseline, 3, 6 and 12 months postenrollment.
6. Graft failure will be defined as return to chronic dialysis, transplant nephrectomy, retransplantation or death. The study coordinator capturing clinical event data will review the medical record at monthly intervals to determine if a study subject has developed graft failure. The timing and cause of each graft loss will be recorded for comparative analysis. Patient death will also be captured in a similar fashion, with timing and cause recorded as well.
7. Healthcare encounters will be defined as any direct encounter (face-to-face) between the study patient and a physician or advance practice provider occurring within a licensed healthcare facility and occurring during the 12-month study. These encounters will be categorized as ambulatory clinic visits, ambulatory procedure visits, acute care/emergency room visits, and hospitalizations. Hospitalizations will be defined as an admission to a hospital with at least one overnight stay. Length of stay within the hospital for readmissions will also be captured. Healthcare encounters will be captured through direct study subject interviews with patients at bimonthly intervals. The study coordinator will record all healthcare encounters that have occurred. If the patient has a health care encounter outside of the study institution, the research coordinator will document the type of encounter to estimate costs, as detailed below.
8. Costs associated with care will be assessed based on data from hospital accounting at the study institution, once the study is completed. Costs will be measured from the time of randomization up until the end of the 12-month follow-up period. Analyses will include all costs associated with inpatient and outpatient care, including hospitalizations, ambulatory care visits, ambulatory procedure visits, acute care/emergency room visits, and laboratory assessments. Costs uniquely associated with the intervention group will include the costs of the devices and data plan provided to the patients; time necessary for training patients on use of the technology; and research pharmacist time associated with the intervention. Total costs will be calculated for each cohort. These data will be electronically captured by providing a list of patients’ medical record numbers to hospital accounting after the completion of the study to allow for accurate and complete billing information to accrue. Costs associated with healthcare encounters that occur outside the study institution will be estimated by acquiring information from the patient regarding the type of encounter and using this data to estimate cost based on cost/charge ratios from the study institution. This will be a cost-consequences analysis, using cost effectiveness methodology, taken from the societal perspective.

## Discussion

Due to the complexities and toxicities associated with their immunosuppressive medication regimens, kidney transplant recipients are at high risk of developing medication safety issues which can lead to hospitalization, increased healthcare expenditures, and ultimately graft loss. Founded on preliminary information, the use of pharmacists and mHealth technology provide a promising and innovative approach to improve medication safety in high-risk patients. The ultimate goal of this research is to demonstrate how patients, pharmacists, and technology can work hand-in-hand to optimize medication-related outcomes and reduce healthcare expenditures.

## Abbreviations

ADE: adverse drug events
DSMP: data safety monitoring plan
GFR: glomerular filtration rate
IRB: institutional review board
ME: medication errors
MNA: medication nonadherence
